# Effect of using different co-ligands during ^99m^Tc-labeling of J18 peptide on SK-MES-1 cell binding and tumor targeting

**DOI:** 10.22038/ijbms.2021.57424.12770

**Published:** 2021-09

**Authors:** Seyed Jalal Hosseinimehr, Soghra Farzipour, Maryam Alvandi, Zahra Shaghaghi

**Affiliations:** 1 Department of Radiopharmacy, Faculty of Pharmacy, Mazandaran University of Medical Sciences, Sari, Iran; 2 Cardiovascular Diseases Research Center, Department of Cardiology, Heshmat Hospital, School of Medicine, Guilan University of Medical Sciences, Rasht, Iran; 3 Department of Nuclear Medicine and Molecular Imaging, School of Medicine, Hamadan University of Medical Sciences, Hamadan, Iran; 4 Department of Nuclear Medicine and Molecular Imaging, Clinical Development Research Unit of Farshchian Heart Center, Hamadan University of Medical Sciences, Hamadan, Iran

**Keywords:** [^99m^Tc ]Tc-HYNIC-(Ser)3-J18, EDDA, HYNIC-SSS-J18, Linker, Lung carcinoma targeting, NSCLC, Tricine

## Abstract

**Objective(s):**

Lung cancer is the main cause of cancer death, and its incidence is increasing worldwide. The goal of this study is to evaluate *in vitro* and *in vivo* tumor targeting behavior of [^99m^Tc]Tc -HYNIC-(Ser)_3_-J18 in lung carcinoma (SK-MES-1)-bearing mice.

**Materials and Methods:**

The J18 (RSLWSDFYASASRGP) peptide was conjugated with hydrazinonicotinamide (HYNIC) via three serine amino acids as a linker at the peptide’s N-terminal and then labeled with technetium-99m using tricine and tricine/EDDA as the co-ligands. The radiolabeled peptides were assessed for *in vitro* receptor binding, specific binding, and saturation affinity. *In vivo* biodistribution studies were also performed for ^99m^Tc-peptide 1 (tricine co-ligand) and ^99m^Tc-peptide 2 (tricine/EDDA coligands) in nude mice bearing SK-MES-1 xenograft tumors.

**Results:**

*In vitro* studies showed high specific binding for ^99m^Tc-peptide 1 in SKMES-1 cells compared with ^99m^Tc-peptide 2 (11.5 vs. 4.5). The K_D_ values for ^99m^Tc-peptide 1 and ^99m^Tc-peptide 2 were reported to be 3.1±0.3 nM and 3.46 ± 0.8 nM, respectively. The biodistribution study also showed high significant tumor to muscle ratios of 5.1 and 6.18 for ^99m^Tc-peptide 1 at 1 and 2 hr after injection, respectively, while these ratios were 3.81 and 5.18 for peptide 2, respectively.

**Conclusion:**

Overall, ^99m^Tc-labeled J18 peptide in the presence of tricine as co-ligand has better *in vitro* and *in vivo* tumor targeting properties in SK-MES-1 cells than tricine/EDDA co-ligands. These findings show that the ^99m^Tc-labeled J18 peptide is a good candidate for lung carcinoma targeting.

## Introduction

Cancer has been found to be a major source of illness and mortality all around the world. Lung cancer was detected in over 2 million instances in 2018, making it the most common type of cancer worldwide (1). Lung cancer is also known as the first major cause of cancer deaths in men, and the second one in women. Lung cancer is generally categorized into small cell (SCLC, 15%) and non-small cell lung carcinoma (NSCLC, 85%). Adenocarcinoma and squamous cell carcinoma are known as the major histological subtypes of NSCLC. A large number of patients are diagnosed at advanced stages of disease in which the prognosis is poor. As a result, morbidity and mortality in these patients are strikingly high. NSCLC treatment options include chemotherapy, radiotherapy, and surgery if they are diagnosed early enough (2). While lung tumors are often initially evaluated with an X-ray or chest CT scan, molecular imaging scans are highly accurate in determining whether a lung mass is cancerous and even eliminating the need for surgical biopsy. Today, positron emission tomography (PET) and single-photon emission computed tomography (SPECT) are two molecular imaging modalities that play increasingly crucial roles in identifying, diagnosing, and treating NSCLC (3). Although PET is superior for staging of NSCLC, it is more expensive and less widely available than SPECT. Radiolabeled peptides with technetium-99m have ideal pharmacokinetics parameters for non-invasive imaging of lung cancer (4). A 15-mer peptide, J18 (RSLWSDFYASASRGP), was discovered by Soendergaard *et al.* in 2014, via the phage display technique. The good tumor targeting of J18 peptide has been reported in some studies (5). In previously conducted surveys, we evaluated ^99m ^Tc-HYNIC-J18 peptide, using tricine/ EDDA mixture and tricine as co-ligands for tumor targeting in non-small cell carcinoma (A-549) xenograft mice model (6, 7). SK-MES-1 is a squamous carcinoma cell line that belongs to the NSCLC subtype (8, 9). Finding a good and ideal tracer capable of especifically targeting SK-MES-1 tumor cells appears to be appealing for detecting lung cancer. This study’s objective is to assess the tumor-targeting efficacy of (^99m^Tc)Tc-HYNIC-(Ser)_3_-J18 with tricine (^99m^Tc-peptide 1) and (^99m^Tc ) Tc-HYNIC-(Ser)_3_-J18 with tricine/EDDA (^99m^Tc-peptide 2) on SK-MES-1 cells and xenograft models.

## Materials and Methods

***Reagents and equipment***

The Pepmic company (China) provided the HYNIC-(Ser)3-J18 conjugate peptide with a purity of 99%. (^99m^Tc) NaTcO4 was obtained by eluting it from an alumina-based Mo-99/Tc-99m generator (Parsisotope, Tehran, Iran). Acetonitrile (HPLC grade) and ammonium acetate were provided from Merck (Darmstadt, Germany). Trifluoroacetic acid (TFA), tin (II)-chloride dihydrate, EDDA, and tricine were obtained from Sigma-Aldrich (St. Louis, MO, USA). Sigma-Aldrich provided trifluoroacetic acid (TFA), tin (II)-chloride dihydrate, EDDA, and tricine (St. Louis, MO, USA). Standard processes were followed to make the solutions, which were made with high-quality water. The radioactivity in the samples was determined using a gamma counter equipped with a NaI (Tl) detector (Delshid, Tehran, Iran). A Knauer HPLC system was used for analytical reversed-phase high-performance liquid chromatography (RP-HPLC) (Germany). The radiolabeled peptides were examined using a Eurospher 100-5, C18, 4.6×250 mm (Knauer, Berlin, Germany) column with a precolumn and a Lablogic mini-scan gamma detector, which was analyzed using Lura image analysis software (Sheffield, UK). RP-HPLC elution was carried out using a solvent system consisting of 0.1 %TFA in acetonitrile (solvent A) and 0.1 % TFA in water (solvent B). A gradient with solvents A and B was conducted as follows: 0 min, 10% A; 0–10 min, 10% –30% A; 10–20 min, 30%–80% A; 20–25 min, and 80%–10% A for a total time of 25 min. The flow rate was set at 1.0 ml min^-1^. Before entering the column, all solvents were filtered and degassed. The Pasture Institute of Iran and the Iranian Biological and Genetic Research Center provided human ovarian cancer (SKOV-3), human breast cancer (MCF7), human lung cancer (SK-MES-1), and adenocarcinomic human alveolar basal epithelial cells (A-549) cell lines (Tehran, Iran). All cell lines were grown in Dulbecco’s Modified Eagle’s medium (DMEM) media supplemented with 10% fetal bovine serum (FBS) and penicillin-streptomycin (Gibco, Grand Island, NY, USA) at 37 ^°^C in a humidified environment with a 5% CO_2_ incubator. 

***Labeling of the HYNIC-(Ser)***_3_ ***-J18 peptide with*** ***technetium-99m***

The radiolabeling procedures for HYNIC-(Ser)_3_-J18 peptide with technetium-99m in the presence of tricine co-ligand and tricine/EDDA mixture co-ligands were conducted in accordance with our group’s prior description (6,7). Briefly, for tricine, 10 μg of HYNIC-SSS-J18 peptide was dissolved in 100 μl of 0.5 M ammonium acetate buffer at pH 6. 50 μl tricine solution (10 mg in 0.5 M ammonium acetate buffer of pH 6) as a co-ligand was added to the HYNIC-SSS-J18 and combined with 100–300 MBq of fresh (^99m^Tc) NaTcO_4_ solution and 40 μg of SnCl_2_.2H_2_O (1 mg/ml in 0.1 N HCl). The mixture was incubated at room temperature for 30 min. For the tricine/EDDA coligand, briefly, 50 μl solution of tricine (20 mg/100 µl in 0.5M ammonium acetate buffer of pH 6.5), 200 μl solution of EDDA (5 mg/200 µl in 0.5M ammonium acetate buffer of pH 6.5), 40 μl solution of SnCl_2_.2H2O (1 mg/ml in 0.1 N HCl) and 100–300 MBq solution of the freshly eluted ^99m^Tc-pertechnetate were added to 10 μl solution of peptide (1 mg/ml in water) in a microtube and incubated at 95 ^°^C for 10 min. The efficacy of radiolabeled peptides was analyzed with RP-HPLC. Thin-layer chromatography (TLC) was used to detect the presence of colloidal reduced hydrolyzed technetium (RHT) using an acetonitrile-water (50%) solvent as mobile phase. 

***In vitro evaluation of radiolabeled peptides***

*Specific Binding Assay*

The *in vitro *receptor binding of the^ 99m^Tc-labeled peptide was evaluated using SK-MES-1 a human lung squamous cell carcinoma, SKOV-3 human ovarian carcinoma, and A-549 human lung adenocarcinoma epithelial and MCF-7 human breast cancer cell lines. All cell lines were kept at 37 ^°^C in a 5% CO_2_ incubator and were maintained in Dulbecco’s DMEM-high glucose supplemented with 10% FBS. Cells were plated in 24-well plates at a density of 4×10^5^ cells/well in 1 ml of complete medium and incubated for 24 hr to allow in cells to settle. Following a wash with cold serum-free media or PBS, 40 nM radiolabeled peptides in the full medium were given to the cells, which were then incubated at 37 ^°^C for 2 hr. Finally, cells in culture plates were trypsinized and released, and radioactivity in the cells was measured. To determine specific versus nonspecific binding, SK-MES-1 cells were seeded into 12-well plates at a density of 5×10^5^ cells/well and incubated with unlabeled peptides at a concentration 500-fold higher than the labeled peptide for 20 min at 37 °C, followed by the addition of the radiolabeled peptide to the wells. These cells were incubated for 2 hr at 37 ^°^C in a humidified incubator. 

*Affinity calculation*

 The affinities of (^99m^Tc) Tc-HYNIC-peptides were determined utilizing a saturation binding experiment with cultivated cells *in vitro* for determination of the dissociation constant as K_D _and the number of binding sites per cell (Bmax). SK-MES-1 cells were incubated for 2 hr at 37 ^°^C with increasing concentrations of (^99m^Tc) Tc- HYNIC- peptides (0.1, 0.5, 1, 2.5, 5, 25, and 50 nM). One dish served as a blocking sample, and it was immersed in a blocking solution containing a 500-fold excess of the non-labeled peptide. For each concentration, three dishes were utilized. Following incubation, the media was withdrawn, and the cells were trypsinized and collected for Gamma counter activity measurement. Prism 5 software (Ver. 5.0) was used to examine the particular binding data using nonlinear regression. 

***In vivo evaluation of radiolabeled peptide***

*Biodistribution in normal mice*

All animal investigations were approved by Mazandaran University of Medical Sciences Research and Ethical Committee in Sari, Iran. Twelve normal female NMRI mice (20–30 g) were randomly divided into three groups of four. 1 μg of (^99m^Tc) Tc –HYNIC–J18 peptide in 100 μl of buffer (18.5 MBq) was injected into the tail vein. All animals were sacrificed one, four, and twenty-four hours following radiolabeled peptide injection. Mice were euthanized with an appropriate dose of ketamine/xylazine (Sigma, USA) diluted with sterile water (1:3 v/v). Following deep anesthesia, blood was extracted by heart puncture with a 1-mL syringe washed with diluted heparin. Blood, liver, spleen, salivary gland, stomach, kidney, muscle, and bone samples were dissected and weighed, and radioactivity was measured. Except for the intestines, which were computed as a percentage of the injected dose per gram tissue (percent ID/g), the tissue uptake data was calculated as a percentage of the administered dose per total sample. 

***Biodistribution in SK-MES-1 xenografted nude mice***

The tumor biodistribution and targeting properties of the radiolabeled peptides were carried out in female C57 nu/nu mice bearing SK-MES-1 (Pasture Institute, North Branch and Amol, Iran). The tumor was grafted by subcutaneous injection of SK-MES-1 cells at a concentration of 4×10^6^ cells/mouse into the right front flank of the mice and left to grow over a period of 20 days. The mice were ready for biodistribution when tumor size reached 0.7–1 cm^3^ in mean diameter, all mice received intravenous co-injections of (^99m^Tc) Tc-HYNIC-(Ser)_3_-J18 peptide 1 or (^99m^Tc )Tc-HYNIC-(Ser)_3_-J18 peptide 2 (1 μg in 100 μl buffer) after 1, 2, and 4 hr post-injection, respectively (n=4 mice per group). In order to measure the radiopeptides’ nonspecific uptake of the radiopeptides, one group of four animals was injected with 300 μg of non-labeled peptide in a 50 μl buffer. The mice in the blocking group were euthanized 2 hr after the injection. Mice organ and tissue samples were collected, and radioactivity uptake was calculated as a percentage of the administered dose per gram tissue (%ID/g).

***Statistical analysis***

All statistical analyses for biodistribution mean and standard deviation estimations were done in Microsoft excel. Unpaired two-tailed t-tests were used for statistical analysis. *P*-values of 0.05 were deemed significant.

## Results

***Radiolabeling***

**Figure 1:**
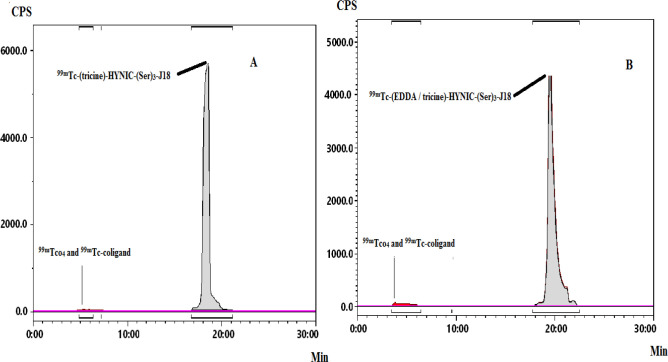
RP-HPLC chromatographs ^99m^Tc-(tricine)-HYNIC-(Ser)_3_-J18 after labeling (A) and ^99m^Tc-(EDDA/tricine)-(Ser)_3_-HYNIC-J18 (B)

HYNIC-conjugated peptide performed efficiently with technetium-99m using tricine or tricine/EDDA mixture as co-ligands. The labeling yield of both radiolabeled conjugates was >99%. The HPLC radiochromatograms showed a single peak, with a retention time of 18–20 and 18-21 min for ^99m^Tc-peptide 1 and ^99m^Tc- peptide 2, respectively (Figure 1). The specific activity for ^99m^Tc-peptide 1 was 18-38 GBq per mg and for ^99m^Tc-peptide 2 it was 43.4 GBq per mg peptide.

***Specific binding assay***

**Figure 2:**
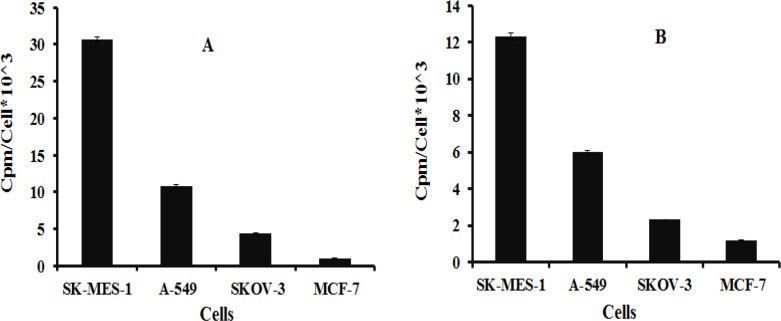
*In vitro* receptor binding of ^99m^Tc-labeled J18- peptide with tricine coligand (A) and tricine/EDDA coligand (B)

**Figure 3:**
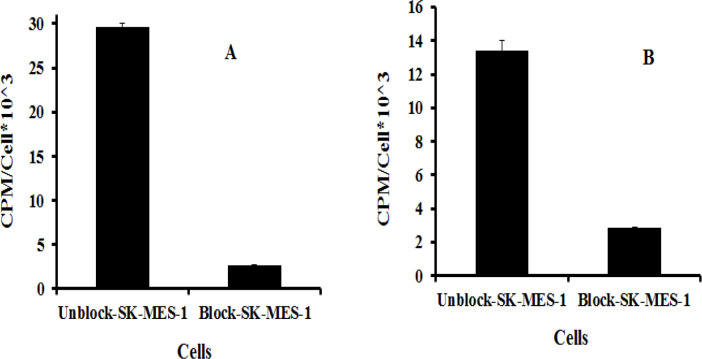
*In vitro* specific binding of ^99m^Tc-(tricine)-HYNIC-(Ser)_3_-J18 (A)and ^99m^Tc-(EDDA/tricine)-(Ser)_3_-HYNIC-J18 (B) on SK-MES-1 cells. Blocking experiment was performed with unlabeled J18 peptide

*In vitro* receptor binding studies (Figure 2) revealed that ^99m^Tc-peptides 1 and 2 had the strongest cellular affinity for the SK-MES-1 cell line. On four cell lines, the order of cellular binding for both radiolabeled peptides was the same (SK-MES-1> A-549>SKOV-3> MCF-7). The receptor binding affinity (as CPM/Cells value) of radiolabeled J18 peptide with tricine co-ligand was 2.5 fold higher than tricine/EDDA as coligand. *In vitro s*pecificity analysis also showed that pre-saturation of SK-MES-1 receptors with unlabeled-J18 peptide reduced nearly 11.5 fold the binding of ^99m^Tc-peptide 1. However, this reduction for ^99m^Tc-peptide 2 was 7.5 fold (Figure 3).

***K***_D_ ***calculation***

**Figure 4:**
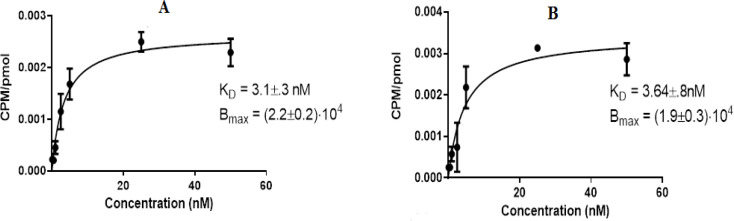
Saturation binding assay curves of ^99m^Tc-(tricine)-HYNIC-(Ser)_3_-J18 (A) and ^99m^Tc-(EDDA/tricine)-(Ser)_3_-HYNIC-J18 (B) on SK-MES-1 cells. K_D_ and Bmax were calculated using Prism software

Saturation binding assays were used to assess the binding of (^99m^Tc) Tc-HYNIC-J18 to the SK-MES-1 cell line (Figure 4). The K_D_ values for ^99m^Tc-peptide 1 and ^99m^Tc-peptide 2 were 3.1±0.3 nM and 3.64±0.8 nM, respectively, indicating that these labeled peptides had a high binding affinity for SK-MES-1. In SK-MES-1 cells, the Bmax was (2.2±0.2)×10^4^ (binding sites/cell) for tricine and (1.9±0.3) ×10^4^ (binding sites/cell) for EDDA/tricine. These findings are consistent with previous research (Table 1). 

***Biodistribution in normal mice***

The biodistribution result of (^99m^Tc)Tc-(Ser)_3_-J18 peptide for both co-ligand systems in normal mice after 1, 4, and 24 hr intravenous injection are presented in Table 2. The high radioactivity levels of both radiolabeled peptides were observed in the kidneys. Muscle, spleen, salivary glands, and blood uptake for both co-ligands were insignificant. In addition, a low radioactivity level was observed for the stomach and salivary glands indicating that the *in vivo* sodium pertechnetate release was minimal, confirming the in *vivo* stability of both radiolabeled peptides

***Biodistribution in SK-MES-1 xenografted nude mice***

To investigate the *in vivo* tumor targeting behavior of (^99m^Tc)Tc-(Ser)_3_-J18 peptide, biodistribution and blocking studies were performed on SK-MES-1 xenografts mice. All biodistribution data are summarized in Table 3. As shown in Table 2 in both radiolabeled peptides, the highest activities were observed in kidneys at all post injection times. The low activities were observed in muscle and blood at all post injection times. The tumor-to-blood ratios of ^99m^Tc-peptide 1 were 5.36, 6.38, and 3.81 at 1, 2, and 4 hr, respectively, while these values were 4.26, 2.96, and 2.09 for ^99m^Tc-peptide2, respectively. The highest tumor-blood ratio was seen for ^99m^Tc-peptide1 at 2 hr. The tumor-to-muscle ratios of ^99m^Tc-peptide 1 were 5.10, 6.16, and 5 at 1, 2, and 4 hr, respectively, while these values were 3.81, 5.18, and 3.28 for ^99m^Tc-peptide 2, respectively. The highest tumor-muscle ratio was observed for ^99m^Tc-peptide 1 at 2 hr. *In vivo* blocking studies with excess non-labeled peptides resulted in significantly reduced 2-h-uptake of both radiolabeled peptides (*P*<0.05). Tumor uptake was decreased from 0.5±0.03 to 0.29±0.05 %ID/g for ^99m^Tc-peptide 1 and from 0.86±0.05 to 0.43±0.05 %ID/g ^99m^Tc-peptide 2. In contrast, radiotracer uptake in other organs was not significantly influenced by the presence of excess non-labeled peptides.

**Table 1:** Overview of affinity binding ^99m^Tc-HYNIC-(Ser)3-J18 on SK-MES-1 vs A-549 cell line

**Type of cell line**	**SK-MES-1**		**A-549**	
**Co-ligand**	**Tricine**	**tricine/EDDA**	**Tricine**	**tricine/EDDA**
**KD( nM)**	**3.1±0.3**	**3.64±0.8**	**4.1 ± 1.3**	**4.4 ± 0.8**
**Bmax( binding sites /cell) **	**(2.2± 0.2)×10** ^4^	**( 1.9 ± 0.3)×10** ^4^	**( 1.8 ± 0.2)×10** ^4^	**(6.2 ± 0.3)** ** 10**^4^

**Table 2:** Biodistribution studies of the complex ^99m^Tc-HYNIC-J18 (tricine and tricine/EDDA) peptides in healthy female mice at 1, 4, and 24 hr after injection

**(%ID/g ± SD)**^a^						
	**Peptide 1** ^b^			**Peptide 2** ^c^		
**Tissue/organ**	**1 hr**	**4 hr**	**24 hr**	**1 hr**	**4 hr**	**24 hr**
**Blood**	**7.60±1.99**	**2.63±0.98**	**0.84±0.34**	**2.42±1.29**	**0.25±0.08**	**0.04±0.01**
**Heart**	**3.23±1.23**	**2.17±1.16**	**0.82±0.48**	**0.98±0.45**	**0.28±0.21**	**0.19±0.07**
**Lung**	**6.31±1.80**	**4.60±3.66**	**1.53±0.75**	**1.65±0.73**	**0.39±0.20**	**0.08±0.06**
**SG&T** ^d^	**3.05±0.91**	**3.41±2.79**	**0.83±0.13**	**1.18±0.63**	**0.47±0.39**	**0.12±0.08**
**Liver**	**3.99±2.76**	**4.61±1.91**	**1.46±0.58**	** 1.13±0.40**	**1.04±0.63**	**0.26±0.07**
**Spleen**	**2.58±1.03**	**2.24±1.81**	**0.81±0.45**	**0.69±0.23**	**00.50±0.31**	**0.17±0.09**
**Kidney**	**29.62±9.43**	**18.97±6.60**	**4.98±2.08**	**6.41±1.71**	**6.96±2.34**	**0.84±0.20**
**Stomach**	**5.58±1.24**	**3.33±1.91**	**0.77±0.20**	**1.17±0.36**	**0.56±0.19**	**0.32±0.14**
**Muscle**	**2.73±0.59**	**1.86±0.86**	**0.56±0.28**	**0.85±0.56**	**0.27±0.20**	**0.06±0.04**
**Bone**	**5.16±0.98**	**2.97±1.94**	**1.64±0.71**	**1.13±0.55**	**1.25±0.83**	**0.46±0.27**
**Intestine** ^e^	**5.70±2.36**	**5.85±2.14**	**1.11±0.38**	**3.76±1.93**	**3.63±1.80**	**0.59±0.29**

^a^ Values are expressed as percentage of injected dose per gram of organ or tissue (mean±SD, n=4); ^b^ Peptide 1: ^99m^Tc-(tricine)-HYNIC-(Ser)_3_-J18; ^c^ Peptide 2: ^99m^Tc-(EDDA/tricine)-(Ser)_3_-HYNIC-J18; ^d^ SG &T: salivary glands and thyroid; ^e^ Data for intestines with contents are presented as percentage of injected dose per whole sample. *P<*0.001

**Table 3:** *In vivo* biodistribution of ^99m^Tc-HYNIC-J18 (tricine and tricine/EDDA) peptides in nude mice bearing SK-MES-1 tumors

**(%ID/g ± SD)** ^a^								
	**Peptide 1** ^b^				**Peptide 2** ^c^			
**Tissue/organ**	**1 hr**	**2 hr**	**4 hr**	**2h-blocked**	**1 hr**	**2 hr**	**4 hr**	**2h-blocked** ^d^
**Blood**	**0.18 ±0.09**	**0.07±0.01**	**0.05 ±0.01**	**0.36 ±0.02**	**0.34±0.01**	**0.29±0.01**	**0.11±0.08**	**0.29±0.02**
**Heart**	**0.19±0.01**	**0.12±0.04**	**0.07 ±0.01**	**0.18 ±0.03**	**0.43±0.04**	**0.18±0.04**	**0.06±0.02**	**0.25±0.03**
**Lung**	**0.19±0.01**	**0.12±0.03**	**0.04 ±0.01**	**0.16 ±0.02**	**0.53±0.05 **	**0.37±0.08**	**0.04±0.04**	**0.42±0.02**
**SG&T** ^e^	**0.23±0.02**	**0.13±0.05**	**0.03 ±0.02**	**0.17 ±0.02**	**0.48±0.04 **	**0.16±0.03**	**0.03±0.03**	**0.28±0.02**
**Liver**	**0.27±0.05**	**0.2 ±0.09**	**0.07± 0.02**	**0.23 ±0.04**	**0.58±0.02**	**0.27±0.01**	**0.06±0.05**	**0.35±0.04**
**Spleen**	**0.25±0.07**	**0.15±0.04**	**0.03 ±0.01**	**0.2 ±0.01**	**0.23±0.02**	**0.18±0.04**	**0.03±0.09**	**0.2±0.01**
**Kidney**	**5.06± 0.05**	**2.50±0.11**	**0.27± 0.03**	**3.20 ± 0.02**	**2.16±0.18**	**1.13±0.01**	**0.26±0.02**	**1.90±0.02**
**Stomach**	**0.38±0.09**	**0.25±0.03**	**0.03 ±0.01**	**0.3 ±0.02**	**0.58±0.02**	**0.24±0.05**	**0.13±0.07**	**0.32±0.02**
**Muscle**	**0.19 ±0.05**	**0.08±0.05**	**0.04 ±0.01**	**0.23 ±0.03**	**0.38±0.04**	**0.17±0.04**	**0.07±0.05**	**0.26±0.03**
**Bone**	**0.13±0.08**	**0.03±0.00**	**0.01 ±0.00**	**0.07 ±0.01**	**0.37±0.03**	**0.21±0.05**	**0.01±0.00**	**0.31±0.01**
**Tumor**	**0.97±0.03**	**0.5±0.03**	**0.2 ±0.09**	**0.29±0.05**	**1.45±0.01**	**0.86±0.05**	**0.23±0.09**	**043±0.05**
**Intestine** ^f^	**0.73±0.06**	**0.59±0.02**	**0.04±0.01**	**0.24 ±0.09**	**0.78±0.01**	**0.53±0.11**	**0.03±0.01**	**0.23±0.09**
**Tumor/organ ratio**								
**Tumor/blood**	**5.36**	**6.38**	**3.81**	**0.8**	**4.26**	**2.96**	**2.09**	**1.48**
**Tumor/muscle**	**5.10**	**6.16**	**5**	**1.26**	**3.81**	**5.18**	**3.28**	**1.65**
**Tumor/bone**	**7.46**	**16.6**	**20**	**4.14**	**3.91**	**7.04**	**23**	**1.38**
**Tumor/liver**	**3.59**	**2.5**	**2.85**	**1.26**	**2.5**	**3.18**	**3.85**	**0.65**
**Tumor/kidney**	**0.19**	**0.2**	**0.74**	**0.09**	**0.67**	**0.76**	**0.88**	**0.22**

^a^ Values are expressed as percentage of injected dose per gram of organ or tissue (mean ± SD, n=4); ^b^ Peptide 1: ^99m^Tc-(tricine)-HYNIC-(Ser)_3_-J18; ^c^ Peptide 2: ^99m^Tc-(EDDA/tricine)-(Ser)_3_-HYNIC-J18; ^d^ To block tumors, mice were pre-administered excess of non-labeled peptide (300 μg) at 0.5 hr before injection of radiolabeled peptide (n=4); ^e^ SG &T: salivary glands and thyroid; ^f^ Data for intestines with contents are presented as percentage of injected dose per whole sample. *P<*0.001

## Discussion

NSCLC is the most prevalence type of lung cancer, early diagnosis and accurate staging are critical parameters for determination of the best possible therapeutic option. Development of molecular imaging agents targeting specific indicators of NSCLC could offer a more precise and sensitive tool for distinguishing lung carcinoma from other benign abnormalities (10, 11). Technetium-99m is a very cost-effective and available diagnostic radiotracer for SPECT (12-15). Peptides, in particular, are one-of-a-kind instruments with exceptional properties for radiolabeling with technetium-99m. Radiolabeled peptides are a new class of pharmaceuticals that have been used in molecular imaging (16). Despite the benefits, the problem with peptides is that they have a limited biologic half-life due to rapid proteolysis in plasma by endogenous peptidases and proteases (16, 17). Molecular modifications of peptides can prevent them from enzymatic degradation (16). To optimize the pharmacokinetic properties of the radiolabeled peptide, we inserted three serine amino acid residues as a spacer group at the N-terminus of J18 (18-20). Additionally, HYNIC was chosen as a bifunctional chelating agent bounded to serine residues in the N-terminal of the peptide. In this work, tumor-targeting characteristics of the J18 peptide bearing two different co-ligand systems were investigated concurrently in both *in vitro* and *in vivo* experiments. This study’s objective was to compare the tumor-targeting behavior of both radiolabeled J18 peptides in the SK-MES-1 lung tumor. Consistent with our previous studies, the high radiochemical purities of both radiolabeled peptides were obtained (>99%). The high specificity and good receptor-binding affinity of ^99m^Tc-peptide 1 and ^99m^Tc-peptide 2 were obtained from SK-MES-1 cells. Two available experiments used for comparison of ^99m^Tc-peptide 1 and ^99m^Tc-peptide 2 for tumor targeting were: i) The *in vitro* assays in which specific binding of radiolabeled peptides to tumor cells determines the receptor binding affinity and ii) The *in vivo* study that was used for pharmacokinetics and *in vivo* tumor targeting of the radiolabeled peptides in a xenografted animal model. It should be noted that cellular specific binding of the radiolabeled peptide is a critical step for its biological assessment. In this specific binding experiment, both radiolabeled peptides in the SK-MES-1 cell line exhibited specific binding to the surface of the SK-MES-1 cell line that was higher as compared with other cell lines such as A-549, SKOV-3, and MCF-7 cells. It is clear that radiolabeled J18 peptide is more favorable than other cell lines such as A-549 as a NSCLC cell that was investigated in our previous study (6, 7). Pre-saturation of SK-MES-1 surface receptors with non-labeled J18 peptide reduced specific binding of J18 peptide nearly 11.5-fold with tricine co-ligand and 7.5 fold with tricine/EDDA co-ligands. It is clear that the ^99m^Tc-J18 peptide with tricine co-ligand showed higher specific binding to SK-MES-1 in comparison with tricine/EDDA coligand. In saturation studies, the radiolabeled J18 was bound to the surface cell with a K_D_ of 3.1 nM and 3.46 nM, which lies near the K_D_ of 4.1 nm and 4.4 nM previously reported for labeled peptide J18 for A-549 cell. This minor variance could be attributable to various cancer cell types which may affect cellular affinity. ^99m^Tc-J18 peptide has shown strong affinity and specificity towards a particular target, even in the absence of knowledge of an appropriate biomarker. Notably, the creation of imaging agents for detection and diagnosis of cancer requires cancer-specific binding of radiolabeled peptides (21). A biodistribution study is a traditional approach for measuring radiolabeled peptide uptake in various organs at various intervals after injection. The biodistribution patterns of J18 peptides (tricine and tricine/EDDA) in normal mice organs were so similar that a significant level of radioactivity accumulation was found in the kidneys. High tumor to muscle radioactivity ratio results in high-resolution images in tumor targeting verification. Besides, fast radiotracer blood clearance causes background low uptake and increased tumor imaging contrast (22). In this study, tumor to normal organ ratios were substantially greater for tricine in comparison with tricine/EDDA coligands. ^99m^Tc-labeled J18 peptide showed better *in vivo* tumor targeting in the SK-MES-1 tumor in comparison with the A-549 tumor. The blocking experiment also showed the specificity of the radiolabeled peptide with reduction of tumor accumulation in xenografted mice pretreated with excess non-labeled peptide. In addition, by comparing the *in vitro* and in *vivo* data from our previous studies, both radiolabeled peptides showed favorable *in vitro* and *in vivo* results in the SK-MES-1 cell line compared with the A549 cell line (6, 7). This result may be due to variation in gene expression between two cell lines (23, 24). As has been shown previously, several detailed comparative studies have established that using different coligands can exert profound effects on the stability, protein binding, receptor binding, affinity, pharmacokinetics, and tumor uptake of the small ^99m^ Tc-labeled peptides.(6, 7, 15, 25). Collectively, radiolabeled J18 peptide with tricine coligand showed favorable *in vitro* and *in vivo* results in the SK-MES-1 cell line in comparison with the tricine/EDDA. These results show that the radiolabeled J18 peptide is a good radiotracer for lung tumor targeting and is favorable for both human lung squamous cell carcinoma (SK-MES-1) and human lung adenocarcinoma epithelial (A-549) tumors. The target antigen or receptor is currently unknown. Therefore, further studies are required to confirm the mechanism of action of the radiolabeled peptide on a unique antigen. However, identification of the peptide receptor and mechanism of action will aid in optimization of the targeting agent.

## Conclusion

 In this study, J18-HYNIC, a 15-mer peptide, was labeled by technetium-99m with tricine or tricine/EDDA as coligands and evaluated for tumor targeting in NSCLC SK-MES-1. Tumor targeting of radiolabeled J18 peptide results were compared with the results of our previous study where the tumor targeting of this peptide in A-549 cells, another NSCLC subtype, was reported. To sum up, radiolabeled J18 peptide with tricine co-ligand showed favorable *in vitro* and *in vivo* tumor targeting properties in two different lung carcinoma cell lines (SK-MES-1 & A-549). The tumor targeting property of the J18 peptide in SK-MES-1 cells was a little higher than in the A-549 cells.

## Authors’ Contributions

SJH, ZS Study conception and design; ZS, MA, SF Data processing, collection, perform experiment; Analysis and interpretation of results: SJH, ZS, SF, MA Draft manuscript preparation; ZS, SF, MA critical revision of the paper; SJH Supervision of the research; SJH, SF, MA, ZS Final approval of the version to be published. 

## Data Availability Information

 All data are available upon request.

## Ethics

This study and all procedures performed were approved by the Ethics Committee of Mazandaran University of Medical Sciences, Mazandaran, Iran.

## Conflicts of Interest

The authors confirm that they no conflicts of interest.
